# Risk Factors and Interpretation of Inconclusive Endoscopic Ultrasound-Guided Fine Needle Aspiration Cytology in the Diagnosis of Solid Pancreatic Lesions

**DOI:** 10.3390/diagnostics13172841

**Published:** 2023-09-01

**Authors:** Renáta Bor, Béla Vasas, Anna Fábián, Mónika Szűcs, Zsófia Bősze, Anita Bálint, Mariann Rutka, Klaudia Farkas, Tibor Tóth, Tamás Resál, Péter Bacsur, Tamás Molnár, Zoltán Szepes

**Affiliations:** 1 Department of Internal Medicine, Albert Szent-Györgyi Medical School, University of Szeged, H-6725 Szeged, Hungary; fabian.anna@med.u-szeged.hu (A.F.); bosze.zsofia@gmail.com (Z.B.); balint.anita@med.u-szeged.hu (A.B.); rutka.mariann@med.u-szeged.hu (M.R.); farkas.klaudia@med.u-szeged.hu (K.F.); toth.tibi89@gmail.com (T.T.); resaltamas@gmail.com (T.R.); bacsurp@gmail.com (P.B.); molnar.tamas@med.u-szeged.hu (T.M.); szepes.zoltan@med.u-szeged.hu (Z.S.); 2 Department of Pathology, Albert Szent-Györgyi Medical School, University of Szeged, H-6725 Szeged, Hungary; vasas.bela@med.u-szeged.hu; 3 Department of Medical Physics and Informatics, Albert Szent-Györgyi Medical School, University of Szeged, H-6720 Szeged, Hungary; szucs.monika@med.u-szeged.hu

**Keywords:** EUS-FNA, pancreatic cancer, risk of malignancy, inconclusive cytology

## Abstract

Background: The inconclusive cytological findings of endoscopic ultrasound-guided fine needle aspiration (EUS-FNA) remain a major clinical challenge and often lead to treatment delays. Methods: Patients who had undergone EUS-FNA sampling for solid pancreas lesions between 2014 and 2021 were retrospectively enrolled. The “atypical” and “non-diagnostic” categories of the Papanicolaou Society of Cytopathology System were considered inconclusive and the “negative for malignancy” category of malignancy was suspected clinically. We determined the frequency and predictors of inconclusive cytological finding. Results: A total of 473 first EUS-FNA samples were included, of which 108 cases (22.83%) were inconclusive. Significant increases in the odds of inconclusive cytological findings were observed for lesions with a benign final diagnosis (OR 11.20; 95% CI 6.56–19.54, *p* < 0.001) as well as with the use of 25 G FNA needles (OR 2.12; 95% CI 1.09–4.01, *p* = 0.023) compared to 22 G needles. Furthermore, the use of a single EUS-FNA technique compared to the combined use of slow-pull and standard suction techniques (OR 1.70; 95% CI 1.06–2.70, *p* = 0.027) and less than three punctures per procedure led to an elevation in the risk of inconclusive cytology (OR 2.49; 95% CI 1.49–4.14, *p* < 0.001). Risk reduction in inconclusive cytology findings was observed in lesions between 2–4 cm (OR 0.40; 95% CI 0.23–0.68, *p* = 0.001) and >4 cm (OR 0.16; 95% CI 0.08–0.31, *p* < 0.001) compared to lesions ≤2 cm. Conclusions: The more than two punctures per EUS-FNA sampling with larger-diameter needle (19 G or 22 G) using the slow-pull and standard suction techniques in combination may decrease the probability of inconclusive cytological findings.

## 1. Introduction

Endoscopic ultrasound (EUS) has recently become a key modality in the identification of solid pancreatic lesions and in the distinction of their benign and malignant origin; furthermore, it facilitates the therapeutic decision-making in terms of the precise staging and determination of resectability. The guideline of the European Society of Gastrointestinal Endoscopy (ESGE) recommends EUS-guided fine needle aspiration (FNA) as a first-line sampling technique for suspected solid pancreatic neoplasms [1,2]. Numerous clinical trials and meta-analyses have demonstrated the efficacy and safety of this procedure, but the inconclusive (low cellularity or the presence of atypical cells of undetermined significance due to technical problems, bloodiness or other artifacts of the smears) cytological results still remain a major challenge in daily practice, since they do not allow a definitive differential diagnosis between benign or malignant condition [3,4,5,6]. In this situation, the re-evaluation of the pathology slides and surgery may also be considered in addition to repeated EUS-FNA sampling [1,7]. Nevertheless, inconclusive cytology often leads to delays in treatment and increases the burden of the patients and the medical costs due to repeated interventions.

The ideal solution to this problem would be to reduce the proportion of EUS-FNA samples with inconclusive results as much as possible. Therefore, the aim of our retrospective study was to assess the frequency and examination- and patient-related risk factors of these findings, as well as the clinical outcome of patients after the EUS-FNA sampling of solid pancreatic lesions regarding risk of malignancy.

## 2. Materials and Methods

### 2.1. Patient Enrollment and Determination of Subgroups

This is a retrospective, single-center cohort study which was carried out in one of the Hungarian tertiary-level referral gastroenterology centers in cooperation with the pathology department. The MedSolution hospital information system was used to collect the medical documentation of patients. All consecutive patients were enrolled between January 2014 and December 2021 who underwent EUS-FNA sampling for solid pancreatic lesions. The patients were divided into two subgroups based on the diagnostic value of the obtained EUS-FNA samples: subgroups of conclusive and inconclusive cytology. We considered inconclusive those cytological results which did not help to establish a definitive diagnosis or to reliably differentiate between the benign or malignant origin of the lesion. For the objective definition of these cases, we used the Papanicolaou Society of Cytopathology System for Reporting Pancreaticobiliary Cytology (PSC system), which facilitates interpretation of the findings by providing information on the evaluability of the sample and the certainty of the malignant diagnosis [8]. All cytology cases classified in the “non-diagnostic” (I) and “atypical” (III) categories of the PSC system were included in the group of inconclusive cases, regardless of the nature (benign or malignant) of the solid pancreatic lesion suggested by the EUS image. Furthermore, selected cases of the “negative for malignancy” (II) category were also added to this group if malignancy was suspected based on the EUS image due to the necessity for further diagnostic steps for validation of the diagnosis. The “neoplastic: benign” (IVa), “neoplastic: other” (IVb), and “malignant” (VI) categories of the PSC system were classified into the conclusive cytology subgroup, as well as the “suspicious for malignancy” (V) category, due to its high risk of malignancy (ROM) in an appropriate clinical setting [9,10].

### 2.2. Objectives of the Study and Clinical Validation of Cytological Findings

Two different primary objectives and one secondary objective were established in our study. Primary objectives were:

(1) To determine the frequency and predictors of inconclusive cytological finding of the first pancreatic EUS-FNA sampling;
(2) To determine the outcome of disease in patients with inconclusive cytology results.

The secondary objective was the identification of clinical factors that influence the ROM of EUS-FNA sampling. These were determined in relation to patients irrespective of the number of EUS-FNA samplings performed during the study period. In assessing the predictors for the inconclusive cytological results, only the characteristics of the first EUS-FNA sample of the patient were evaluated to avoid possible bias due to the repetition of cases/factors. The predictors were identified by assessing the effect of patient-related (age, gender, location, and size of lesion, benign, or malignant final diagnosis) and procedure-related factors (investigator, size of needle, number of punctures per procedure, biliary stent placement prior sampling, diagnosis based on EUS image) on the two outcomes.

The ROM was determined based on the final diagnosis given at the end of patients’ follow-ups, which was made in one of the following modalities: (i) conclusive repeated biopsy finding which could be obtained by repeated EUS-FNA, US-guided transabdominal biopsy, endoscopic biopsy of tumor invading the upper gastrointestinal tract, etc., (ii) surgical intervention (macroscopic morphology and/or histological examination); (iii) autopsy finding; and (iv) clinical course of the disease (in malignant cases—tumor progression or metastasis formation; in cases of inflammation—regression by imaging modalities or response to treatment, etc.). The clinical course of the disease was assessed after a follow-up of at least one month, except for those patients who had a clearly tumor-related death within less than one month due to rapid cancer progression (although no autopsy was performed). The ROM was defined by the number of malignant cases divided by total number of cases within each category of clinical predictors. The efficacy data of EUS-FNA examinations were determined by the comparing the cytological findings with the final diagnosis. False-positive cases were defined as benign lesions which were incorrectly diagnosed by cytology as malignant. Similarly, all cases were considered false-negatives if a malignant neoplasm was incorrectly diagnosed by cytology as benign. Inconclusive “non-diagnostic” (I) and “atypical” (III) categories were considered as the absence of malignancy, so they were classified as a true-negative in the case of a benign final diagnosis and a false-negative in malignant cases.

### 2.3. EUS-FNA Procedure and Pathological Evaluation

EUS-FNA samplings were performed by two experienced endoscopists (Z.Sz. or L. Cz.) using linear echoendoscope (Olympus GF-UCT 140; Olympus GF-UCT 160; Olympus Optical, Tokyo, Japan) and 19 G, 22 G, and 25 G FNA needles (Echotip Ultra; Cook Ireland Ltd., Limerick, Ireland; EZ Shot 2 and 3, Olympus Optical, Tokyo, Japan). The punctures were performed using 5 or 10 mL continuous standard suction (SS) and/or slow-pull (SP) techniques with the same needle during approximately 7–10 back-and-forth movements performed in a fanning manner under continuous ultrasound control. The number of punctures, the suction force, and the size of the needle were not uniform, and depended on the endoscopist’s preference and the characteristics of the lesion due to the retrospective nature of the study. The obtained material from the needle was pushed on the slides with the reinsertion of the stylet, from which the grossly visible coherent pieces of tissue were removed and placed in a tube filled with 10% buffered formalin (formalin-fixed, paraffin-embedded (FFPE) cell blocks) without macroscopic on-site evaluation (MOSE). The direct smears were made from the remaining specimen and fixed in 96% methanol for at least 10 min. Samples were prepared by EUS nurses or by the gastroenterologists assisting the endosonographer. Rapid on-site evaluation (ROSE) was unavailable. All cytological smears and FFPE were stained by hematoxylin-eosin (HE); immunocytochemistry was performed in most FFPE tissues and in select cases on smears with high cellularity.

### 2.4. Ethics Approval and Consent to Participate

This study was approved by the Regional and Institutional Human Medical Biological Research Ethics Committee of the University of Szeged, Hungary (ethics approval number: 3680/2015 SZTE). All the included patients have signed an informed consent form for the scientific use of their medical data. The study was carried out in accordance with the Declaration of Helsinki.

### 2.5. Statistical Analysis

Statistical analysis was performed with R statistical software version 3.6.0 (R Foundation, Vienna, Austria) and with SPSS software version 28 (SPSS Inc., Chicago, IL, USA); *p* values of less than 0.05 were considered significant. Descriptive statistics were expressed as means and medians with ranges. Logistic regression model, Pearson Chi-squared, and Fisher’s exact tests were applied to identify the clinical factors that can modify the incidence of inconclusive cytology and that can influence the ROM of pancreatic lesions.

## 3. Results

### 3.1. Characteristics of Patients and EUS-FNA Samplings

**Figure 1:**
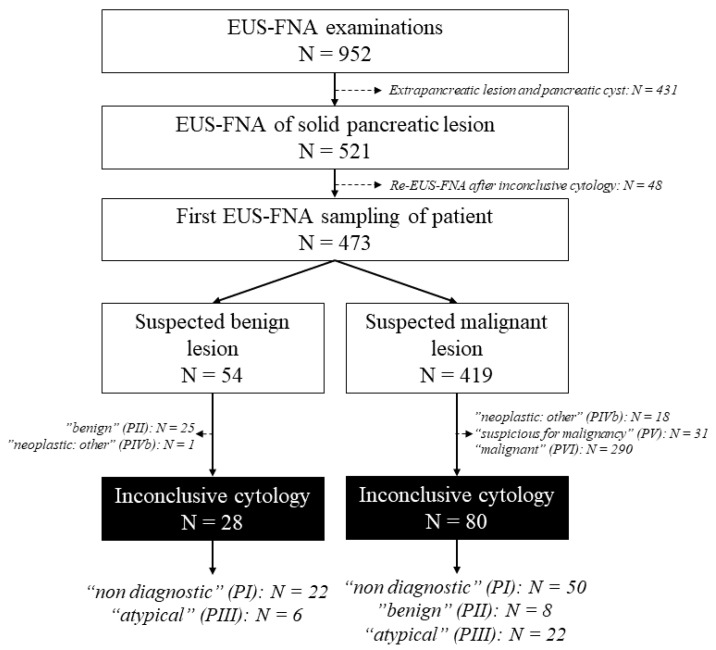
Patient enrollment in the study.

**Figure 2:**
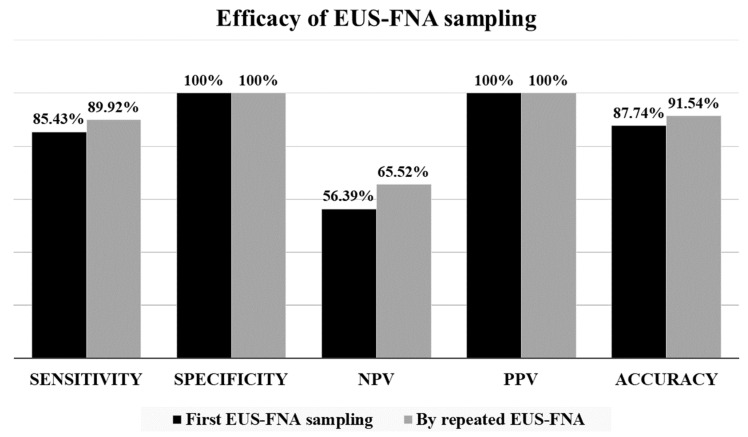
Efficacy of the EUS-FNA sampling of solid pancreatic lesions.

A total of 473 patients with solid pancreatic lesion were enrolled who had undergone 521 EUS-FNA examinations during the study period: in forty-four cases two samplings and in two cases three samplings were performed. For each patient, we assessed the outcome data from the first sampling. (Figure 1) Based on the EUS image, the endoscopist presumed the lesion was malignant in 419 cases (88.58%) and benign in 55 cases (11.63%). Most lesions were localized to the pancreatic head and uncinate process (*n* = 322, 68.08%) with a mean diameter of 33.83 ± 14.18 mm (range 5–90 mm, median 30 mm). (Table 1) Cytological examination confirmed a definite neoplastic etiology (“malignant” (VI), “suspicious for malignancy” (V), and “neoplastic: other” (IVb) categories) in 340 cases (71.88%) and only 33 samples (6.98%) were classified as being in the “negative for malignancy” (II) category of the PSC system. There were no cases classified as “neoplastic: benign” (IVa) in the study cohort. In contrast, at the end of the mean follow-up of 13.77 months (range 0.1–106.4 months, median 5.67 months), the rate of neoplastic lesions was lower, namely 83.51%, of which 392 cases (82.88%) were malignant and 3 cases (0.63%) were neoplastic benign. The final diagnosis was validated histologically in 185 cases (39.11%), while in 288 patients (60.89%) the diagnosis was confirmed by the clinical course of the disease with a mean follow-up period of 10.54 months (range 0.1–106.4 months, median 2.0 months). The histologic specimens included 45 small biopsy samples with EUS-FNA or other modalities (24.32%), 107 surgical excision or resection specimens (57.84%), and 33 autopsy samples (17.84%). The sensitivity, specificity, and diagnostic accuracy of patients’ first EUS-FNA sampling were 85.43%, 100.00%, and 87.74%, respectively, which increased to 89.92%, 100.00%, and 91.54% by repeated EUS-FNA of non-diagnostic cases. (Figure 2) In 36 out of 46 cases, repeated EUS-FNA sampling was sufficient to establish the diagnosis. EUS-FNA sampling-related complications were recorded in five cases, which included one case of iatrogenic duodenal perforation, one case of gastrointestinal bleeding, one case of acute pancreatitis, and two cases of asymptomatic amylase elevation. Errors in the cytological diagnosis were identified in five cases. Two ductal adenocarcinomas were incorrectly diagnosed as neuroendocrine tumors (NET), while in two cases, severe reactive abnormalities accompanying chronic pancreatitis complicated by acute inflammation were falsely interpreted as malignancies. Furthermore, one adenocarcinoma was initially reported as low-grade intraductal papillary mucinous neoplasm (IPMN) due to presumable peritumoral sampling.

### 3.2. Frequency and Predictors of Inconclusive Cytological Findings

The first EUS-FNA sampling of patients provided inconclusive results in 108 cases (22.83%), but there was no substantial fluctuation in the proportion of these cases over the study period. This rate varied between 16.67% and 25.58% over the years. Two examiners performed EUS-FNA sampling at our institute for whom there was no significant difference in the efficacy of sampling and in the proportion of inconclusive cases (21.26% vs. 27.20%, *p* = 0.176). (Table 2) Inconclusive samples were obtained more frequently for lesions smaller than 2 cm (43.42%) compared to lesions between 2–4 cm (23.35%, *p* = 0.001) and larger than 4 cm (10.71%, *p* < 0.001).

The use of the 19 G needle proved to be the most advantageous, but the difference compared to 22 G needles was not statistically significant (OR 0.35, 95% CI [0.08–1.01], *p* = 0.088). In contrast, the use of 25 G needles was associated with substantially higher odds of inconclusive findings (OR 2.12, 95% CI [1.09–4.01], *p* = 0.023). The combined use of SP and SS techniques within a single EUS-FNA intervention reduced the proportion (20.34%) and risk (OR 1.70, 95% CI [1.06–2.70], *p* = 0.027) of inconclusive cytology findings compared to the use of a single technique (30.25%). When comparing each technique using the combined method, a significant difference was detectable only in the case of SP (31.51%; OR 1.80, 95% CI [1.02–3.12], *p* = 0.038). The use of three to four punctures per examination seems to be the most advantageous. Increasing the number of punctures did not reduce the risk of inconclusive findings (OR 0.90, 95% CI [0.45–1.71], *p* = 0.763). However, fewer than three punctures elevated the risk of inconclusive results (OR 2.49, 95% CI [1.49–4.14], *p* < 0.001). The mean number of smears obtained per puncture had no influence on the rate of inconclusive results (*p* = 0.674). Furthermore, in our cohort, without the use of MOSE, EUS-FNAs that resulted in both direct smears and FFPE were not associated with a reduction in the rate of inconclusive cytology compared to samplings resulting in direct smears only (26.32% vs. 22.53%, *p* = 0.594). The presence of a biliary stent did not increase the risk of inconclusive results (OR 1.08, 95% CI [0.67–1.71], *p* = 0.748). The rate of successful EUS-FNA sampling was higher after metal stent implantation (absence of stent: 77.58%, plastic stent: 74.11%, metal stent: 83.87%), but the difference was not statistically significant (*p* = 0.420).

The inconclusive results showed the strongest correlation with benign origin of the lesion determined by the end of follow-up, where their rate was 65.38% compared to 14.43% as seen in malignant cases (OR 11.20 CI 95% [6.56–19.54], *p* < 0.001). This may also be due to the high rate of non-evaluable, particularly bloody, or cell-poor smears (“non-diagnostic” I) obtained when sampling benign lesions, significantly more often than in malignant lesions (47.44% vs. 8.86%, *p* < 0.0001). Further reason for this may be that the smears with intact acinar cells or mild inflammatory abnormalities (“benign” II) may raise the possibility of peritumoral sampling if cross-sectional imaging and/or EUS images suggest suspicion of malignancy. When examining the effect of localization on the diagnostic value of sampling, we found that abnormalities in the pancreatic tail were associated with a remarkably low rate of inconclusive cases (6.67%) compared to other localizations (head 27.06%, uncinate process 25.37%, and body 20.00%, respectively).

Multivariate analysis confirmed the influence of four predictors on inconclusive findings: pancreas tail localization (OR 0.13 CI 95% [0.03–0.42], *p* = 0.002), lesion size greater than 4 cm (OR 0.24 CI 95% [0.10–0.54], *p* = 0.001), and malignant EUS morphology (OR 0.11 CI 95% [0.02–0.38], *p* = 0.002) were associated with a decrease in risk, whereas the benign origin of the lesion (OR 56.97 CI 95% [17.40–272.78], *p* < 0.001) led to an increase in risk.

### 3.3. Outcome Patients with Inconclusive Cytology Results

At the end of the follow-up period, 57 cases (52.78%) in the inconclusive subgroup were found to be malignant. The final diagnosis was based on a histopathological examination (repeated EUS-FNA *n* = 7; transabdominal US-guided biopsy *n* = 19, surgical sample *n* = 24; autopsy *n* = 7) in 57 cases (52.78%), while in the remaining 51 cases (47.22%) it was determined by the clinical course of the disease during a mean follow-up period of 20.50 months (range 0.23–106.4 months, median 8.92 months). In 25 of these patients, the endosonographic image was suggestive for benign disease and no lesion was detected during the follow-up EUS examination, requiring repeated sampling. These histologically unidentified benign lesions were chronic pancreatitis (*n* = 12), acute necrotizing pancreatitis (*n* = 3), and autoimmune pancreatitis (*n* = 1), furthermore, in nine cases, disappearance of the lesion was noted using cross-sectional imaging and/or EUS during follow-up. In 13 of the 26 cases, when the endosonographic morphology was suspicious for malignancy, benign disease was presumed based on the results of the cross-sectional imaging and/or repeated EUS examination (focal lesion disappeared during follow-up *n* = 7; chronic pancreatitis *n* = 3; acute necrosing pancreatitis *n* = 2; pseudocyst *n* = 1). The re-biopsy of 13 patients with a rapidly progressive underlying disease and deteriorating general condition was waived due to lack of clinical relevance, because they were no longer suitable for oncological treatment or refused it.

### 3.4. Clinical Factors Influencing the ROM

In the study cohort, the overall ROM of the EUS-FNA was 83.51% regardless of the EUS diagnosis which warranted the FNA sampling. The ROM for females and males was 88.11% and 78.60% (*p* = 0.006), while for the age groups below 60 years, between 60 and 75 years, and over 75 years, the risk was 73.08%, 86.87%, and 85.45%, respectively. The mean age of the patients with a malignant final diagnosis was significantly higher compared to patients with a benign diagnosis (67.4 ± 10.9 years vs. 62.4 ± 15.1 years, *p* = 0.001). Lesion size had a significant correlation (*p* < 0.001) with ROM because the abnormalities smaller than 2 cm were more often benign (39.47%) compared to lesions between 2–4 cm (13.62%) and larger than 4 cm (9.29%). (Table 3) Elevated CA19-9 (>27 U/mL) and CEA (>4.7 ng/mL) values above normal were also found more frequently in malignant cases (89.55% and 91.18%, *p* < 0.001). The “non-diagnostic” (I) category showed no difference in the proportion of benign and malignant lesions at the end of follow-up (48.61% vs. 51.39%), whereas the “atypical” (III) category had a high ROM of 75.00%. The inconclusive subgroup included only those cytological specimens in the “negative for malignancy” (II) category, in which malignancy was suspected based on EUS imaging; nevertheless, by the end of follow-up malignancy was confirmed in only 11.11% of cases. These values were even more pronounced when the entire study population was evaluated: the ROM for the “negative for malignancy” categories was 3.03%. Within the inconclusive subgroup, only one case judged to be benign using the EUS image had a final diagnosis of benign (ROM 3.57%), whereas the ROM for the EUS image suggestive of malignancy was 70.00%. These values were also slightly more explicit when we evaluated the entire population: ROM in benign EUS morphology was 3.70% compared to 93.79% for the ROM seen in malignant EUS images (*p* < 0.001).

In the subgroup of inconclusive cytological findings, coexistence of the identified predictors leads to a further increase in ROM, so, in the “atypical” (III) category, for lesions with malignant EUS morphology larger than 2 cm, the ROM achieved WAS 94.74%. In contrast, in the “non-diagnostic” (I) and “negative for malignancy” (II) categories, the ROM for lesions smaller than 2 cm was only 25.93%, which decreased further for CA19-9 in the normal range (ROM 0.00%) and for benign EUS morphology (ROM 0.00%).

## 4. Discussion

In our study, we retrospectively reviewed the data of EUS-FNA samplings in our tertiary-level referral gastroenterology center to provide guidance for the interpretation of inconclusive cytological findings and to help further therapeutic decision-making. The advantage of our study is that it was carried out in close collaboration with experienced cytologists in the department of pathology and the PSC classification system for solid pancreatic tumors was routinely applied during the study period to facilitate interdisciplinary communication. EUS-FNA sampling was performed by one of the two endosonographers and the cytological samples were assessed by at least one of the three experienced pathologists, and in challenging cases by two of them. The small number and similar level of expertise of doctors involved in the evaluation allowed for the elimination of interobserver variability. The greatest limitation of our study is its single-center retrospective cohort nature, which resulted in the restricted availability of clinical data on patients’ symptoms (abdominal pain, jaundice, weight loss, etc.), and tumor marker findings (CEA, CA19-9, CgA). The gastroenterological evaluation of pancreatic lesions and EUS-FNA samplings were performed at our institute as a tertiary-level referral medical center, however, the patients’ follow-up was frequently performed in primary- or secondary-level medical institutions, which limited the availability of these data. Additionally, confirmatory cytological and/or histological sampling was performed in only a small number of patients, so the definitive diagnosis was determined mainly based on the behavior of the disease during follow-up.

The diagnostic accuracy achieved in our study is consistent with the results published in international studies and high-quality meta-analyses, with a pooled sensitivity of 84–89% and a specificity of 96–99% [3,11,12]. However, the diagnostic efficacy of the method for solid nodules in chronic pancreatitis are significantly lower: sensitivity 65% (52.6–75.6%) and specificity 96.8% (75–99.7%), respectively [13]. Despite the convincing data, the NPV of EUS-FNA for suspected pancreatic tumors is considered low, and in our study it was also barely above 50%; furthermore, the inconclusive (atypical cells, suspicious for malignancy), negative for malignancy, or nondiagnostic results do not allow the definitive diagnosis of the benign conditions. In this situation, further cross-sectional imaging, another sampling, or even surgery may be necessary to shorten the diagnostic delay. Our data also show that repeated EUS-FNA sampling substantially increases the diagnostic accuracy of EUS-FNA from 87.74% to 91.54%. This is consistent with the meta-analysis published in 2020 by Lisotti et al. which also demonstrated that repeated EUS-FNA for the diagnosis of solid pancreatic masses in cases of a previous nondiagnostic or inconclusive result is an effective diagnostic tool, with 77% (66–86%) sensitivity, 98% (78–100%) specificity, 99% (98–100%) PPV, and 61% (60–63%) NPV [14]. The additive benefit of rapid onsite evaluation (ROSE) was also shown by a significant increase in sensitivity (85% vs. 65%) and a reduction in the number needed to diagnose (1.2 vs. 1.7). The need for ROSE is not yet discussed in detail in the ESGE guidelines, and there is conflicting evidence in the scientific literature [1,2,15,16,17,18]. However, recent studies have emphasized its benefits in terms of both diagnostic yield and cost-effectiveness [19,20,21]. Furthermore, a multicenter retrospective study by de Moura et al. concluded that FNB alone produces a similar diagnostic yield compared to EUS-FNA with ROSE. Therefore, FNB may be useful in cases where the results of previous EUS-guided sampling were indeterminate [22]. An alternative solution is the use of self-ROSE, where a specimen’s adequacy is immediately assessed by a trained endoscopist [23]. In our study, only EUS-FNA samplings were evaluated, excluding EUS-FNB samplings, but ROSE was not available, and we have no experience with the self-ROSE technique.

In our study, larger needle diameters were associated with a decrease in the rate of inconclusive cytological findings. This contradicts the results of a meta-analysis published in 2019, in which no significant difference was observed between the 22 G and 25 G needles used during EUS-FNA in the diagnosis of solid pancreatic lesions based on randomized trials [24]. Although most retrospective cohort studies have shown no difference in the efficacy of conventional needles of 19 G, 22 G, and 25 G diameter, in recent years, data from several clinical trials have been published that found the inferiority of conventional needles compared to new types of FNA and FNB needles. The recently developed Franseen needles were superior to EUS-guided sampling with a conventional needle with respect to diagnostic accuracy, particularly in patients who required immunostaining [25]. The novel fork-tip FNB needles were also found to be superior to FNA needles in term of proportion graded as a straightforward diagnosis (69% vs. 51%) and median pathology viewing time (188 vs. 332 s; *p* < 0.001) [26]. These two FNB needle types achieved the highest degree of cellularity in a single biopsy, with a diagnostic accuracy greater than 90% [27]. Furthermore, the FNB needles also outperformed FNA needles in the sampling of pancreatic and nonpancreatic lesions in terms of diagnostic accuracy (87% vs. 80%, *p* = 0.02) and tissue core rate (80% vs. 62%, *p* = 0.002) [28].

In addition to the type of needle applied, the technique of EUS-FNA sampling may also influence the outcome of the sampling. In our study, the combined use of the SP and SS techniques in a single examination, along with 3–4 punctures per sampling, resulted in the lowest proportion of inconclusive cytology findings. It should be pointed out that the fanning technique was applied for all EUS-FNA cases irrespective of the suction force, since previous studies have already demonstrated its superiority over the standard approach [29]. A guideline on the technical aspects of the EUS-FNA was published in 2017 and has not been updated yet [30]. It recommends the use of 10 mL standard suction for the EUS-guided sampling of solid masses with 25 G or 22 G FNA needles, however, the results of recently published prospective and retrospective clinical trials have questioned this [31,32,33]. A meta-analysis published in 2023, which compared the efficacy of SP, dry-suction, modified wet-suction, and no-suction techniques has found that the modified wet-suction provided the highest rate of sample adequacy; furthermore, dry suction was associated with significantly higher rates of blood contamination as compared with the SP technique (OR 1.44 95% CI [1.15–1.80]) [34]. The number of punctures required to achieve the optimal diagnostic accuracy of EUS-FNA is still unclear [35]. The ESGE guideline recommends a performance of three to four needle punctures with an FNA needle or two to three punctures with an FNB needle when ROSE is unavailable [30]. In contrast, the white paper of the American Gastroenterological Association (AGA) also considers the size of the lesion when making recommendations based on clinical trials: in the absence of ROSE, optimally four punctures should be performed to achieve highest diagnostic accuracy in pancreatic solid lesions >2 cm in size and at least six punctures in lesions <2 cm [36,37]. Studies published in recent years have reported varying conclusions regarding the effect of pancreatic tumor size on the diagnostic yield of EUS-FNA. Uehara et al. highlighted that EUS-FNA was accurate in the evaluation of suspected pancreatic malignancies regardless of the size and location of lesion [38]. However, in another study, this accuracy was only achievable when ROSE was available [39]. Similar to several other studies, we have also verified that the size of the lesion influences the outcomes of EUS-FNA [37,40,41]. In our study, the rate of inconclusive cytological results was significantly increased for lesions smaller than 2 cm. The lower rate of inconclusive results in larger lesions may be explained by the fact that they are easier to identify and less frequently sampled peritumorally. Another important reason for this may be the high ROM of these lesions, and our study has also pointed out that the most important predictor of inconclusive cytological findings is a benign final diagnosis. However, it should also be considered that in cases of large lesions the risk of formation of necrotic areas within the tumor is higher, as cells obtained from these locations are unsuitable for the establishment of a diagnosis. Furthermore, the sampling of especially vascularized areas could be also disadvantageous due to massive blood contamination obscuring tumor cells on the smears. Contrast-enhanced EUS-FNA (CE-EUS-FNA) sampling can eliminate these problems by providing detailed, visualized information on the blood perfusion of the lesion, thus avoiding necrotic or particularly vascularized areas. Significantly higher efficacy was achieved by CE-EUS-FNA compared to conventional EUS-FNA sampling in cases of pancreatic lesions: pooled diagnostic accuracy was 88.8% (85.6–91.9%) vs. 83.6% (79.4–87.8%), respectively [42]. The stiffness of the tumor and the degree of fibrosis can also affect the effectiveness of sampling, as it is assumed that the aspiration of cells of hard, fibrotic cancers requires a sampling technique using greater suction power [43]. Both pancreatic carcinomas and chronic pancreatitis are typically hard lesions due to prominent desmoplastic stromal reactions. A retrospective study performed by Togliani et al. found that the adequacy of EUS-guided tissue acquisition was negatively affected by the presence of fibrosis (OR 8.37 CI 95% [2.33–30.0]), and by the location of the lesion in the head/uncinate process (OR 0.37 CI 95% [0.14–0.99]) [44]. However, the higher presence rate and grading of tissue fibrosis in lesions located in the head/uncinate process seemed to be responsible for the negative impact on sample adequacy. In our study, there was no clear correlation between tumor location and the rate of inconclusive cytology results. The lesions in the pancreatic tail were associated with a significantly lower rate of inconclusive findings (6.67%) compared to other location; however, it is questionable whether this factor can be considered a true predictor of inconclusive cases, since this localization was the least frequent in the study population (*n* = 60) and this group had a low rate of benign lesions (13.33%), lesions smaller than 2 cm (6.67%), and needle diameter of 25 G (1.67%).

In our study, the proportion of conclusive results was not higher in EUS-FNA examinations where both direct smears and FFPE were obtained, compared to those where only direct smears were obtained. This may also be explained by the fact that the formalin-fixed sample was not histologically evaluable in a large proportion of cases; it appeared only as a blood coagulum. Therefore, the proportion of conclusive cytology findings for FFPE was only slightly above 70%. The use of MOSE could be an alternative solution for the assessment of the adequacy of specimens if ROSE is unavailable, potentially enhancing the diagnostic yield of FFPE. However, during the study period in our institute, MOSE was not implemented [45,46]. A prospective pilot study by Iwashita et al. determined that that the ideal cut-off value of the length of the macroscopically visible core on MOSE, indicating the presence of a histologic core specimen, is ≥4 mm. This achieved a sensitivity of 93.1% and specificity of 72.0% [47]. One of the most significant advantages of FFPE, as opposed to direct smears, is that it provides tissue architectural information in addition to cytomorphology. Moreover, it is compatible with a wide range of molecular and immunohistochemical techniques. Immunohistochemistry often proves essential for the differential diagnosis or prognostic evaluation of tumors, including pancreatic metastases and neuroendocrine tumors [48,49].

There was a very strong correlation between PSC categories, EUS morphological diagnosis, and the ROM. The final diagnosis was 75.00% malignant in the “atypical” (III) category and 3.03% in the “negative for malignancy” (II) category, while the ROM for benign and malignant EUS morphological diagnoses was 3.70% and 93.79%, respectively. This may be explained by the fact that the benign diagnosis is made with the utmost caution by both the gastroenterologist and the pathologist. The pathologists classified cytological findings as “negative for malignancy” (II) in very select cases, (i.) in the complete absence of cellular atypia in correlation with EUS findings negative for neoplastic process and (ii.) in the presence of cytologic features characteristic for specific nonneoplastic lesions, including autoimmune pancreatitis or ectopic spleen. The situation was similar for a diagnosis established based on the EUS image by gastroenterologists. The “non-diagnostic” (PI) cytological findings, however, did not provide any guidance on the choice of further diagnostic steps to be taken and were not related to the ROM. These results are in accordance with international data [50,51]. The systematic review of eight studies by Nikas et al. showed that the ROM of PSC categories varied widely: the ROM of the “non diagnostic” (I), “negative for malignancy” (PII), and “atypical” (PIII) categories were in the ranges of 8–50%, 0–40%, and 28–100%, respectively [52]. The largest meta-analysis, which included 3566 patients from 23 studies, separately assessed the outcomes of atypical cytological findings of EUS-FNA in solid pancreatic masses and found that the ROM of this category was 58% (95% CI 47%–69%) [53]. The presence of a mass and absence of a history of pancreatitis were significant predictors for pancreatic malignancy in cases of the cytological diagnosis of “atypical cells”, while the absence of a mass in the EUS images or history of chronic pancreatitis was more likely to be associated with a benign lesion [54]. The authors are in agreement that institutions (both cytopathologists and endoscopists) should monitor and keep their “atypical” cytology rates low, but there is no consensus recommendation or guideline that defines atypical cytology rate as a quality indicator or determines its minimum standard value. In our institute, the rate of “atypical” (III) PSC category was low, with 5.92%.

## 5. Conclusions

Our retrospective cohort study confirmed the high diagnostic efficiency of EUS-FNA in the sampling of solid pancreatic lesions. A positive diagnosis of EUS-FNA correlates well with the definitive diagnosis of the lesion, while for inconclusive samples, further diagnostic and therapeutic steps are recommended to determine the nature of the disease based on the morphological diagnosis of EUS and the PSC category together. The EUS morphology of lesions showed the closest correlation with ROM, therefore, the endoscopist’s proficiency, the thoroughness of the examination, and the adequate evaluation of lesions (description, image documentation) are of critical importance in the interpretation of inconclusive cases. In the case of EUS morphological signs suggestive for a benign lesion and “negative for malignancy” (PII) cytological findings, a follow-up with the patient may be sufficient; in contrast, repeated sampling is required if malignancy is suspected on the basis of EUS morphology or in the cases of “non-diagnostic” (PI) and “atypical” (PIII) cytological categories. In this case, the EUS-FNA is recommended again, as our results confirmed that the diagnostic value of this method increases significantly with repetition, and the use of a larger diameter needle may be advantageous. To reduce the burden on patients and the healthcare system, in the case of “atypical” (PIII) cytology, clinicopathological consultation and possible revision of smears with a second pathologist should be considered after detailed clinical data of the patient have been provided. The rate of inconclusive EUS-FNA findings can be successfully reduced by using larger diameter needles (22 G and 19 G) and by the combined use of SP and SS techniques within a single intervention. We recommend three or four punctures per sampling: fewer than two punctures increased the proportion of inconclusive cases, whereas more than four punctures did not improve the sampling efficiency.

## Figures and Tables

**Table 1:** Clinical characteristics of patients and EUS-FNA examinations (*n* = 473).

Characteristics of Patients		Characteristics of EUS-FNAs	
Male/female	229/244	Examiners A/B:	348/125
Age (year)	66.63 ± 11.81 (18–95; median: 68)	Mean number of puncture per examination	3.44 ± 1.07
Mean size of lesion (mm)	33.83 ± 14.18	Number of puncture per examination	
Size of lesion		≤2 punctures	90 (53.93%)
≤20 mm	76 (16.07%)	3–4 punctures	311 (14.78%)
20–40 mm	257 (54.33%)	>4 punctures	72 (19.19%)
≥40 mm	140 (29.60%)		
		Mean number of smear pairs per examination	2.11 ± 1.01
Location of lesion			
head	255 (53.91%)		
uncinate process	67 (14.16%)	Sampling technique	
body	90 (19.03%)	only slow-pull (SP)	73 (15.43%)
tail	60 (12.68%)	only standard suction (SS)	46 (9.73%)
diffuse	1 (0.21%)	both SP and SS	354 (74.84%)
Histology of lesion			
Ductal adenocarcinoma	352 (74.42%)	Size of EUS needle	
Primary bile duct carcinoma	2 (0.42%)	19 G	33 (6.98%)
Solid pseudopapillary npl.	3 (0.63%)	22 G	395 (83.51%)
Well-differentiated NET	15 (3.17%)	25 G	45 (9.51%)
Neuroendocrine carcinoma	3 (0.63%)		
Low-grade IPMN	1 (0.21%)	Biliary stent	129 (27.27%)
High grade IPMN (clinical suspicion of malignancy)	2 (0.42%)	Type of lesion based on EUS image	
Myxofibrosarcoma	1 (0.21%)	benign	54 (11.42%)
Hematolymphoid tumor	2 (0.42%)	malignant	419 (88.58%)
Metastatic carcinoma	15 (3.17%)	Cytological finding based on PSC System	
Ancient schwannoma	1 (0.21%)	“non-diagnostic”	72 (15.22%)
Serous cystadenoma	1 (0.21%)	“benign”	33 (6.97%)
Intrapancreatic spleen	1 (0.21%)	“atypical”	28 (5.92%)
Acute necrosing pancreatitis	12 (2.54%)	“neoplastic: other”	19 (4.02%)
Autoimmune pancreatitis	4 (0.85%)	“suspicious for alignancy”	31 (6.55%)
Chronic pancreatitis	31 (6.55%)		
Histologically unverified focal lesion disappeared during follow-up	27 (5.71%)	“malignant”	290 (61.31%)

**Table 2:** Predictors of inconclusive cytological findings (univariable analysis).

	Conclusive *n* = 365	Inconclusive *n* = 108	Odds Ratio (95% CI)	*p* Value
Examiner				
ExA	274 (78.74%)	74 (21.26%)		
ExB	91 (72.80%)	34 (27.20%)	1.38 (0.86–2.20)	0.176
Location of lesion				
Head	188 (73.73%)	69 (27.06%)		
Uncinate process	50 (74.63%)	17 (25.37%)	0.92 (0.48–1.67)	0.781
Body	72 (80.00%)	18 (20.00%)	0.67 (0.37–1.19)	0.187
Tail	56 (93.33%)	4 (6.67%)	0.19 (0.06–0.49)	0.002
Size of lesion				
≤20 mm	43 (56.58%)	33 (43.42%)		
20–40 mm	197 (76.65%)	60 (23.35%)	0.40 (0.23–0.68)	0.001
≥40 mm	125 (89.29%)	15 (10.71%)	0.16 (0.08–0.31)	<0.001
Size of needle				
19 G	30 (90.91%)	3 (9.09%)	0.35 (0.08–1.01)	0.088
22 G	307 (77.72%)	88 (22.28%)		
25 G	28 (62.22%)	17 (37.78%)	2.12 (1.09–4.01)	0.023
Sampling technique				
Both SP and SS	50 (68.49%)	72 (20.34%)		
SP or SS alone	33 (71.74%)	36 (30.25%)	1.70 (1.06–2.70)	0.027
Slow-pull (SP)	83 (69.75%)	23 (31.51%)	1.80 (1.02–3.12)	0.038
Standard suction (SS)	282 (79.66%)	13 (28.26%)	1.54 (0.75–3.02)	0.219
Number of punctures per procedure				
≤2 punctures	56 (62.22%)	34 (37.78%)	2.49 (1.49–4.14)	<0.001
3–4 punctures	250 (80.39%)	61 (19.61%)		
>4 punctures	59 (81.94%)	13 (18.06%)	0.90 (0.45–1.71)	0.763
Type of sample				
Only direct smears	28 (73.68%)	10 (26.32%)	1.23 (0.55–2.54)	0.594
Direct smears and FFPE	337 (77.47%)	98 (22.53%)		
Origin of lesion				
Benign	27 (34.62%)	51 (65.38%)	11.20 (6.56–19.54)	<0.001
Malignant	338 (85.57%)	57 (14.43%)		
EUS morphology				
Malignant	339 (80.91%)	80 (19.09%)		
Benign	26 (48.15%)	28 (51.85%)	4.56 (2.54–8.25)	<0.001
Presence of biliary stent				
Absence	256 (77.58%)	74 (22.42%)		
Presence	109 (76.22%)	34 (23.78%)	1.08 (0.67–1.71)	0.748
Plastic stent	83 (74.11%)	29 (25.89%)	1.21 (0.73–1.97)	0.453
Metal stent	26 (83.87%)	5 (16.13%)	0.67 (0.22–1.66)	0.720

**Table 3:** Risk of malignancy (ROM) in patients with solid pancreatic lesion (univariable analysis). (* Limitation: data on CA19-9 and CEA were only available in 57.08% and 49.89% of patients, respectively).

All Cases *n* = 473	Risk of Malignancy	Odds Ratio (95% CI)	*p* Value
Gender			
Female	88.11%	2.02 (1.23–3.36)	0.006
Male	78.60%		
Size of lesion			
≤20 mm	60.53%		
20–40 mm	86.38%	4.14 (2.31–7.42)	<0.001
≥40 mm	90.71%	6.37 (3.12–13.65)	<0.001
PSC category			
“non-diagnostic” (PI)	48.61%		
“negative for malignancy” (PII)	3.03%	0.03 (0.00–0.17)	0.001
“atypical” (PIII)	75.00%	3.17 (1.25–8.90)	0.020
EUS morphology			
benign	3.70%	0.03 (0.00–0.01)	<0.001
malignant	93.79%		
Tumor markers *			
CA19-9 (n = 270)		5.75 (2.99–11.22)	<0.001
CA19-9 elevation	89.55%		
CA19-9 normal	59.42%		
CEA (n = 236)		3.94 (1.88–9.11)	0.001
CEA elevation	91.18%		
CEA normal	72.39%		

## Data Availability

The datasets generated for this study are available on request from the corresponding authors.
